# Rapid species discrimination of similar insects using hyperspectral imaging and lightweight edge artificial intelligence

**DOI:** 10.1098/rsos.240485

**Published:** 2024-07-31

**Authors:** Xuquan Wang, Zhiyuan Ma, Yujie Xing, Tianfan Peng, Xiong Dun, Zhuqing He, Jian Zhang, Xinbin Cheng

**Affiliations:** ^1^ MOE Key Laboratory of Advanced Micro-Structured Materials, Shanghai 200092, People’s Republic of China; ^2^ Institute of Precision Optical Engineering, School of Physics Science and Engineering, Tongji University, Shanghai 200092, People’s Republic of China; ^3^ Frontiers Science Center of Digital Optics, Shanghai 200092, People’s Republic of China; ^4^ East China Normal University, Shanghai 200241, People’s Republic of China

**Keywords:** hyperspectral imaging, insect species discrimination, convolutional neural network, edge artificial intelligence

## Abstract

Species discrimination of insects is an important aspect of ecology and biodiversity research. The traditional methods based on human visual experience and biochemical analysis cannot strike a balance between accuracy and timeliness. Morphological identification using computer vision and machine learning is expected to solve this problem, but image features have poor accuracy for very similar species and usually require complicated networks that are unfriendly to portable edge devices. In this work, we propose a fast and accurate species discrimination method of similar insects using hyperspectral features and lightweight machine learning algorithm. Feature regions selection, feature spectra selection and model quantification are used for the optimization of discriminating network. The experimental results of six similar butterfly species in the genus of *Graphium* show that, compared with morphological recognition with machine vision, our work achieves a higher accuracy of 92.36 ± 3.04% and a shorter inference time of 0.6 ms, with the tiny-size convolutional neural network deployed on a neural network chip. This study provides a rapid and high-accuracy species discrimination method for insects with high appearance similarity and paves the way for field discriminations using intelligent micro-spectrometer based on on-chip microstructure and artificial intelligence chip.

## 1. Introduction

Insects are the largest group of animals on Earth, with a wide variety and diverse forms [1]. The discrimination of insect species is an important aspect of ecology and biodiversity research [2]. Traditional identification methods of human visual perception primarily rely on the subjective experience of ecologists, resulting in low accuracy rate when distinguishing between similar genus or species [3,4]. Biochemical analysis techniques, such as DNA and rRNA gene sequencing, can provide high identification accuracy, but they are hindered by complex procedures and poor timeliness, owing to the dependence on laboratory equipment [5]. Thus, there is an urgent need for a species discrimination method that can balance both accuracy and timeliness.

With the rapid development of computer vision and digital image processing technology, the methods for automatic recognition of insect species based on image features were proposed and popular in the past few decades [3]. The geometric features, wing shape, wing colour and surface texture of insects have been used as key feature in the research of species classification [3,6]. With traditional statistical methods, researchers proposed the concept of branch length similarity entropy to characterize wing shape features and the concept of grey-level co-occurrence matrix to extract colour and texture features, in the classification of butterflies [6,7]. In recent years, deep learning has extended the species recognition based on image features to more scenarios with a better accuracy, owing to its ability to perform automatic feature extraction [8,9]. Carvajal *et al*. used three pre-trained convolutional neural network (CNN) models on the ImageNet dataset to identify 19 different species of Lepidoptera insects with a recognition accuracy of over 92% [2]. Another research compared the classification performance of 11 deep neural networks on the Indian butterfly dataset and achieved the highest accuracy of 94.44% using ResNet-152 [10]. However, most of the datasets used in these studies are species with significant differences in appearance [2,4,6,11]. For species with high phenotypic similarity, the feature differences extracted by visual models are not obvious, and such differences are often complicated by age, population and seasonal variations, which are easily misclassified [10,12].

Hyperspectral imaging (HSI) technology can simultaneously obtain the geometric appearance and spectral features of targets with the advantages of high efficiency, timeliness and non-destructive detection [13,14]. Nowadays, by combining with deep learning, HSI has been successfully applied in many fields of scientific research and industrial production, including the species identification of insects in ecological study [15–17]. Compared with machine vision and image recognition methods, HSI usually has a higher recognition accuracy owing to the increased dimension of spectral sensing. Some studies have used this technique to study the wing interference patterns and structural colours of butterfly and dragonfly [18–20]. Takahashi evaluated the interspecific differences among 12 fruit flies using the average reflectance spectra of hyperspectral images [21]. Furthermore, some researchers developed a system that can efficiently capture multi-spectral images of Lepidoptera, which will help compare the differences between different species at different classification scales [22]. This research demonstrated the effectiveness of hyperspectral images in interspecies classification and differences in insects evaluation. However, unfortunately, owing to the complex algorithms and large amount of data, the HSI data pretreatment and analysis models are mostly deployed on external computers and physically separated from the hardware of hyperspectral equipment. This separating mode is clearly not a boon to field applications of species discrimination. Therefore, a lightweight HSI artificial intelligence (AI) discriminating model that can be deployed on edge devices, instead of the computing centre, has significant value in ecological and biodiversity research.

In this work, we propose a rapid and accurate species discrimination method for similar insects using HSI and lightweight AI algorithm. As experimental demonstration, six butterfly species with high similarity in the genus of *Graphium* are discriminated by a miniaturized deep learning model, which is deployed on a neural network processing unit (NPU) chip. Feature regions selection, feature spectra selection and model quantification are used for the compression and optimization of discrimination algorithm. The experimental results show that the lightweight network extracted from hyperspectral features achieves higher accuracy with less complexity compared with morphological recognition with machine vision. This study provides a species discrimination method for similar insects with both accuracy and timeliness and paves the way for miniaturized and field-use instrument with on-chip integration of spectroscopic microstructure and AI chip.

## 2. Methodology

The method we proposed is based on a prior law that there is a large amount of data redundancy in hyperspectral images, including both spatial and spectral dimensions [23]. Spatial redundancy comes from the irrelevant and non-feature regions of samples. Spectral redundancy comes from two aspects: one is the high correlation commonly existing between adjacent spectral bands and another is the non-feature spectral bands for specific species, which have no value to analysis model. Thus, the selections based on regions of interest (ROI) and bands of interest (BOI) for specific species are used as prior knowledge for the simplification of hyperspectral data cube, while the network quantification is used for the compression of on-chip discriminating model.

**Figure 1.**
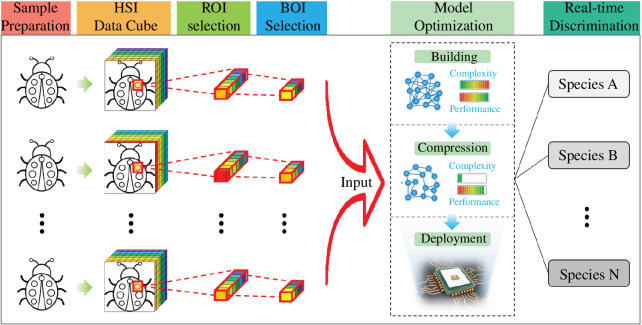
The proposed method of rapid and accurate species discrimination.

As shown in figure 1, the proposed method of species discrimination consists of six steps.

### 2.1. Sample preparation

To remove the influence caused by sample preprocessing, all samples in this work are specimens prepared using the same and standard method.

### 2.2. Hyperspectral imaging data acquisition

The aim of HSI data acquisition is to get reflectivity data cube, and the operations are completely the same as in traditional hyperspectral applications.

### 2.3. Regions of interest selection

To remove the irrelevant and non-feature regions of samples from hyperspectral data cubes, the preprocessing selection of ROIs is used to reduce the data amount. As one of the most important organs, wings play an unparalleled role in species identification of insects. Thus, we propose to select ROIs on wings for further feature extraction and classification. Two principles are mainly considered during the selection of ROI. On the one hand, these areas must be located in the middle of the wings to minimize the impact of sample defects and contamination, while on the other hand, they must have clear spectral features and high reflectivity to ensure classification effectiveness.

### 2.4. Bands of interest selection

We firstly use the Savitzky–Golay (SG) method to smooth the spectra, in order to reduce the noise while preserving the shape and width of the curve [24,25]. And then, the competitive adaptive reweighted sampling (CARS) algorithm is used for the final feature bands extraction [26,27]. Adaptive reweighted sampling was employed to reduce wavelength number in a competitive manner, and the root mean squared error of cross-validation (RMSECV) is calculated as the criterion of optimal feature extraction. In this way, a key-spectra dataset was acquired from the full-spectra dataset, with the same number of spectra and less spectral bands.

### 2.5. Model optimization

We adopt the deep learning model based on CNN to extract sample features for species classification. In the model training stage, the dataset of spectra after ROI and BOI selections is used for model building. In an effort to deploy AI model on a NPU chip for on-the-spot inference and prediction, model quantification and conversion are made for the compression of network at the edge.

### 2.6. Real-time discrimination

Field identifications are executed using on-chip discriminating model.

## 3. Methodology and experimentation

### 3.1. Experimental material

**Figure 2.**
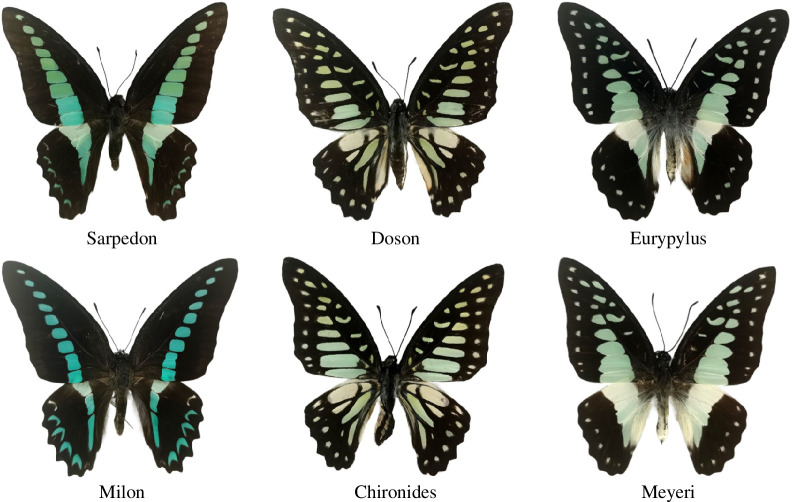
The samples of six butterfly species in the genus of *Graphium*.

Butterflies (Lepidoptera: Rhopalocera) are the second largest group of insects, with approximately 17 000 recorded species on Earth [28]. The species classification and identification of butterflies have been a significant area of research in the field of entomological taxonomy for a long time [3]. In this work, six butterfly species in the genus of *Graphium* are selected for experiments, including *G. sarpedon*, *G. milon*, *G. doson*, *G. chironides*, *G. eurypylus*, and *G. meyeri*. The total number of samples is 140, including 10 samples from *G. meyeri* and 26 samples from each other butterfly species. All the specimens were made using standard method including softening, pinning, stretching wings and drying [29,30]. As shown in figure 2, the boundary shape, spot distribution and colour of the *Graphium* samples all have high similarity. Therefore, fast discrimination between some species of the samples is especially difficult even for professional scientists [5]. In this article, we use the class names of the six butterflies as Sarpedon, Milon, Doson, Chironides, Eurypylus and Meyeri for short.

### 3.2. Hyperspectral images acquisition

**Figure 3.**
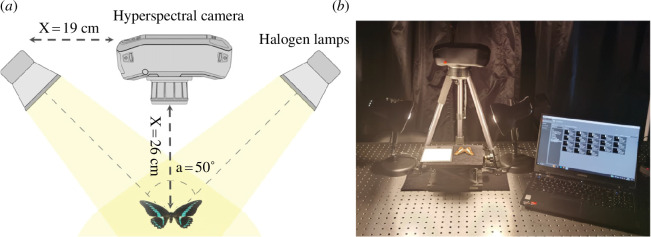
The HSI data acquisition system: (*a*) schematic diagram; (*b*) experimental image.

The HSI system used in this work, as shown in figure 3, consists of three parts: a portable hyperspectral camera mounted on a tripod, an illumination system with two 43 W halogen lamps and a computer for data acquisition. Considering the possibility of field use, we chose a hand-held hyperspectral camera, Specim IQ (Specim Spectral Imaging Ltd), with a wavelength range of 400–1000 nm and a spectral resolution of 7 nm. It is a line scan camera based on push-broom technology with 204 spectral bands. The Specim IQ Studio software is installed on the computer to export hyperspectral data and manage camera settings.

To minimize light scattering errors, the specimens of butterfly and standard white reference (WR) were placed flat on a black background with low reflectance. The camera lens was oriented vertically downward, with a distance of 26 cm from the butterfly specimen. Clear hyperspectral images of the samples were obtained by adjusting a suitable focal length and exposure time; for this work, the exposure time is 20 ms. The resulting hyperspectral images, aside from the actual raw image data of the target area, were further processed to yield reflectance spectral data of every pixel. The reflectivity of the target pixel was acquired according to the equation


(3.1) $$R\;=\;\frac{S_{Pixel}-S_{DBG}}{S_{WR}-S_{DBG}},$$

where $S_{\text{Pixel}}$ and $S_{\text{WR}}$ are the raw signals of the target pixel and WR region, respectively, and $S_{\text{DBG}}$ is the dark signal of the camera to the dark background (DBG).

### 3.3. Discriminating model

With the advantages of high efficiency in adaptive feature learning, CNN has been successfully applied in classification and identification of machine vision and HSI application scenarios [2]. In this work, a discriminating model based on CNN structure is trained, compressed and deployed on the NPU chip of RK3588 for rapid identification.

Firstly, we established a dataset containing 1400 spectral curves using selected spectra by ROI and BOI. According to the ratio of 4 : 1 : 1, all samples of the six species were divided into training set, validation set and test set. In particular, the spectra of the same sample cannot appear in both the training set and testing set simultaneously. In order to evaluate the performance and generalization ability of model accurately, we used the stratified fivefold cross-validation method to process and test the dataset. This method randomly divides the entire dataset into five mutually exclusive subsets and ensures consistency in the proportion of samples for each category in each subset. By sequentially selecting each subset as the test set, we ensure that all data will be used as the test set for model evaluation. Model training was conducted on a Lenovo computer with the GTX 1660 graphical processing unit and 16 GB RAM. The version of deep learning framework was TensorFlow 2.2.0 with Keras 2.3.0. Categorical Cross-entropy Loss and Adam Optimizer with adaptive learning rate were used in the training stage.

Additionally, to compare the effectiveness of the proposed method with traditional deep learning-based machine vision classification, we used the typical networks of ResNet-18 and VGG-11 to classify the RGB image of six species [31,32]. The image dataset was captured using the RGB lens integrated with SpecimIQ, and supplemental samples were generated using data augmentation strategy with geometric and photometric transformations such as rotation, zooming, shifting and colour inversion. In this study, the data augmentation is actualized based on the imgaug library of Python. We obtained a total of 1400 RGB images for all six species, which are also divided into training set, validation set and test set in the same ratio of 4 : 1 : 1. Similarly, the pictures of the same sample cannot appear in both the training set and testing set simultaneously.

Finally, the classification model trained by key-spectra dataset is deployed to the RK3588 series AI chip, which has triple NPU cores with up to six tera operations per second (TOPS) computing power. To further reduce the scale of on-chip inference model, model transformation and quantification were made by the tool RKNN-Toolkit2 1.4.0 installed on Ubuntu 20.04. For binary computing, the data type of INT8 has higher throughput and lower memory requirements than FP32. Our goal of hardware acceleration was to convert models from FP32 to INT8 without significant accuracy loss. Finally, the compressed and optimized RKNN model was loaded for inference by the tool RKNN-Toolkit-Lite2 1.4.0 running on the chip of RK3588.

### 3.4. Model evaluation

We evaluated the model from both performance and complexity dimensions. The performance evaluation metrics include precision (Pr), recall (Re) and F1-score (F1), as illustrated in the following equations [33–35]:


(3.2) $$Pr\;=\;\frac{TP}{TP+FP},$$

(3.3) $$Re\;=\;\frac{TP}{TP+FN},$$

(3.4) $$F1\;=\;\frac{Recall\times Precision}{Recall+Precision},$$

where TP indicates the number of true positives, FP indicates the number of false positives and FN indicates the number of false negatives. The complexity evaluation metrics include floating point operations (FLOPs) and parameters, which were used to evaluate the computational power and memory consumption of the model.

## 4. Results and discussion

### 4.1. Data preprocessing and feature extraction

**Figure 4.**
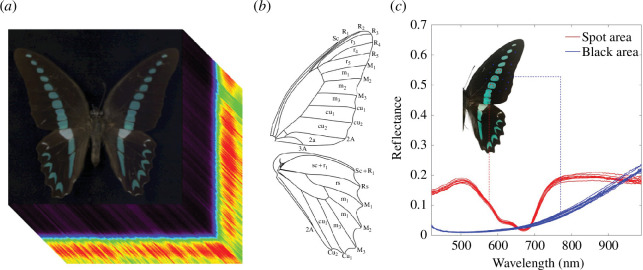
(*a*) Three-dimensional data cube of the spectral image; (*b*) wing veins and compartments of *Graphium* genus; (*c*) the spectra of spot area and black area in the wings of Milon.

The three-dimensional data cube of the spectral image is shown in figure 4*a*, with a size of 512 pixels × 512 pixels × 204 bands. The *x*-axis and *y*-axis are two spatial dimensions, while *z*-axis is the spectral dimension. The regions of the sample and WR were captured into the same data cube, and the spectrum of each pixel was calculated automatically with formula (3.1) by SpecimIQ. Considering that both spectral ends of the camera have a lower signal-to-noise ratio, only the spectral range of 420–990 nm was used in this work, including 191 spectral bands [36].

As shown in figure 4*b*, six species have the same distribution of wing veins and compartments, which were marked in detail. We selected several compartments randomly in the centre of wings for spectra comparison and found the same phenomenon in all species. For a specific species, the spectra in spot position and the black background of wings are significantly different, while the spectra of the same pattern in different compartments are completely identical. As shown in figure 4*c*, this phenomenon is illustrated with the sample of Milon. Owing to the low reflectance of all the samples, it is clear that the spectra of the spot region have more feature information and higher signal quality.

Based on the above analysis, we finally chose the spot region in compartments of r5, m1, m2, m3 and cu1 for both left and right wings as ROIs. We select 3 × 3 pixels for every ROI and take the average as an effective spectrum. Therefore, a total number of 1400 spectra were obtained as our full-spectra dataset for all samples, with 10 spectra for every sample.

**Figure 5.**
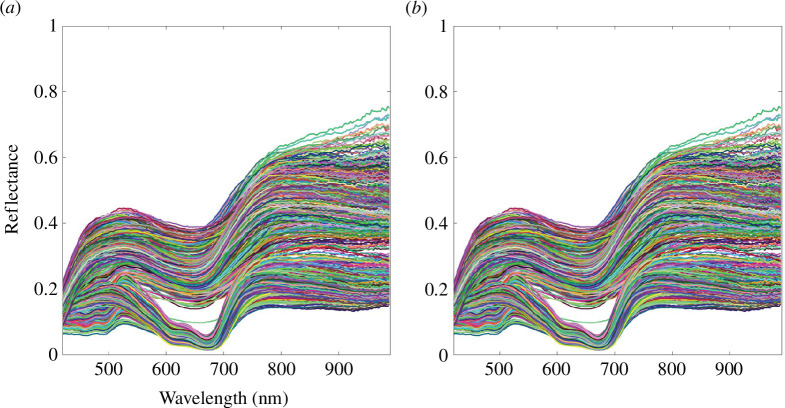
The spectral curves of all samples: (*a*) before SG smoothing; (*b*) after SG smoothing.

Before the feature selection of spectral bands, SG smooth was made to full-spectra dataset, with window points of 5 and a polynomial order of 2 [25]. We named it the SG-full-spectra dataset. The spectral curves before and after smoothing are plotted separately in figure 5*a,b*. We can notice that the SG smoothing operation reduces the noise of spectral curves while obviously preserving the original spectral characteristics.

**Figure 6.**
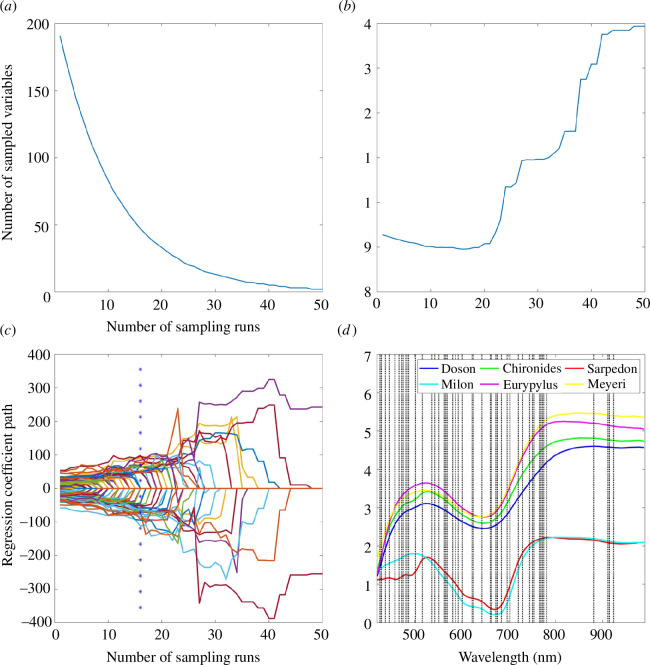
Results of feature wavelengths selection using the CARS algorithm: (*a*) the relation between feature wavelengths and sampling runs; (*b*) the relation between RMSECV and sampling runs; (*c*) the regression coefficients of feature wavelengths; (*d*) the selected feature wavelengths marked on the spectral curves with grey dashed line.

Feature wavelengths were extracted by the CARS algorithm, with a sampling rate of 0.8 [26]. We set the number of sampling runs to 50. As the number of sampling runs increases, figure 6*a,b* illustrates the changes in effective feature wavelengths and RMSECV values. Owing to the existence of an exponentially decreasing function, the wavelength variable initially decreases rapidly and gradually slows down later. The RMSECV values decrease first and then continue to increase. When the number of sampling runs is 16, the RMSECV value reaches the minimum, indicating that the model has reached its optimal state. Figure 6*c* displays the regression coefficient path for each wavelength, with a blue asterisk line marking the sampling run where the minimum RMSECV value occurs. According to the ‘survival of the fittest’ principle, a larger absolute value of the regression coefficient corresponds to a stronger predictive ability. We ultimately chose 47 effective feature wavelengths. As shown in figure 6*d*, the selected feature wavelengths are indicated with grey dashed lines in the average spectral curves of six butterfly species. Thus, we acquired a key-spectra dataset with 47 featured bands from the full-spectra dataset.

### 4.2. Model training results

**Figure 7.**
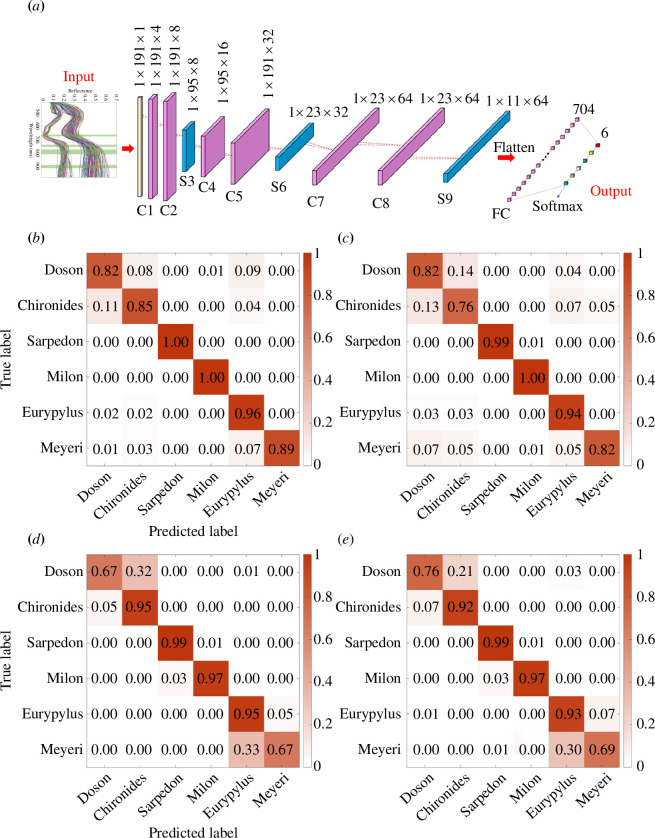
(*a*) The network structure of 1D-CNN-12, consists of input layer, convolution layers C1 and C2, pooling layer S3, convolution layers C4 and C5, pooling layer S6, convolution layers C7 and C8, pooling layer S9, full connection layer FC, and output layer. The discriminating result of 1D-CNN-12 based on (*b*) the SG-full-spectra dataset and (*c*) the key-spectra dataset. The discriminating results of (*d*) ResNet-18 and (*e*) VGG-11 based on RGB images.

As shown in figure 7*a*, we designed a one-dimensional CNN with 12 layers called 1D-CNN-12, including input layer, convolution layers, pooling layers, full connection layer and output layer [37]. The input of 1D-CNN-12 is the spectra with dimensions of (47,1) for key spectra or (191,1) for full spectra. The convolution kernels used in convolution layers C1, C2, C4, C5, C7 and C8 are 4 (with a size of 2), 8 (with a size of 4), 16 (with a size of 3), 32 (with a size of 2), 64 (with a size of 3) and 64 (with a size of 2). The activation function is tanh. Maximum pooling is used in pooling layers S3 (with a size of 2), S6 (with a size of 4) and S9 (with a size of 2). Both convolution layers and pooling layers are set up with padding and a stride of 1. After flattening, the output of 1D-CNN-12 is implemented through a full connection layer with an activation function of softmax. We adopted the dropout method with a rate of 0.3 to prevent over-fitting during the training phase. The epochs are set to 300 with a batch size of 8.

According to the stratified fivefold cross-validation method, we obtained the average accuracy, standard deviation and confusion matrix for five evaluations of different models. The discriminating result of 1D-CNN-12 based on the SG-full-spectra dataset and the key-spectra dataset are shown in figure 7*b,c*, while the discriminating results of ResNet-18 and VGG-11 based on the RGB images dataset are shown in figure 7*d,e*. The classification accuracy of ResNet-18 in the test set is 88.79 ± 7.36%, and most errors occur in the confusions between Doson and Chironides, and between Eurypylus and Meyeri, owing to their similar appearance. Compared with ResNet-18, the classification accuracy of VGG-11 improves to 89.79 ± 5.61%, and the stability of the model has also been further improved. The confusions between Doson and Chironides have been emended. The F1-score for Doson and Chironides is 0.90 and 0.81, respectively, but still not good enough. This indicates that it is difficult to accurately distinguish similar species barely relying on image features. By comparison, the spectral classification model presents impressive results. It performs well in the classification of Eurypylus and Meyeri, with precision of 0.86 and 0.8, respectively. The accuracy of 92.36 ± 3.04% shows the enormous potential of spectral information in species classifications.

The complexity evaluation results are shown in table 1. It can be found that both computation and parameter quantities have decreased by multiple orders of magnitude. The first reason is the changes in data dimensions, one-dimensional convolution with smaller consumption. The second reason is that owing to significant spectral differences, shallower networks can be used for spectra input. The above results demonstrate the feasibility of edge deployment on the chips with low computing power.

**Table 1.** The complexity evaluation results of different models.

model	accuracy	FLOPs	parameters
ResNet-18	88.79 ± 7.36%	3.7G	11.2M
VGG-11	89.79 ± 5.61%	13.4G	128.1M
1D-CNN-12 (SG-full-spectra)	92.36 ± 3.04%	20.1K	20.3K
1D-CNN-12 (key -spectra)	89.64 ± 5.09%	16.7K	16.8K

### 4.3. Model compression and on-chip discrimination

**Figure 8.**
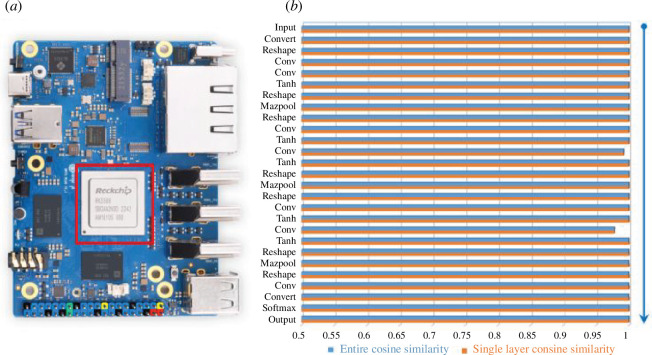
(*a*) The used NPU chip of RK3588. (*b*) The conversion error of each layer and the cumulative error of all layers during quantization.

To demonstrate the feasibility of the proposed method for real-time species identification in the field applications, the model of 1D-CNN-12 was transformed and deployed on the NPU chip of RK3588, as shown in figure 8*a*. We compared the consistency between the deployed network which was on the chip and the original network which was on the computer. The cosine similarity was used to indicate the effect of model compression and quantization, which can represent the conversion error of each layer and the cumulative error of all layers. Figure 8*b* shows the cosine similarity results of both entire model and every single layer, demonstrating that most layers in our conversion have fantastic accuracy with the cosine similarity better than 0.999. Two convolutional operators have slightly lower accuracy, which are 0.992 and 0.978, but the entire cosine similarity is still 1, owing to the correction and adjustment during the model conversion.

With the test set of SG-full-spectra as input, we completed the actual on-chip inference and the discrimination accuracy was 92.36%, which coincides with the result on computer. The inference time of a single spectrum is 0.6 ms with the NPU operating frequency of 1000 MHz. This running result also indicates the feasibility of deploying the model on low-cost processors or microcontroller chips. The above results demonstrate that the proposed method is effective for the balance between accuracy and timeliness in the rapid species discrimination of insects.

### 4.4. Ablation studies

In this section, we conduct ablation studies to verify the effectiveness of the proposed method, including the feature regions selection, the SG smoothing method and the feature spectra selection.

**Figure 9.**
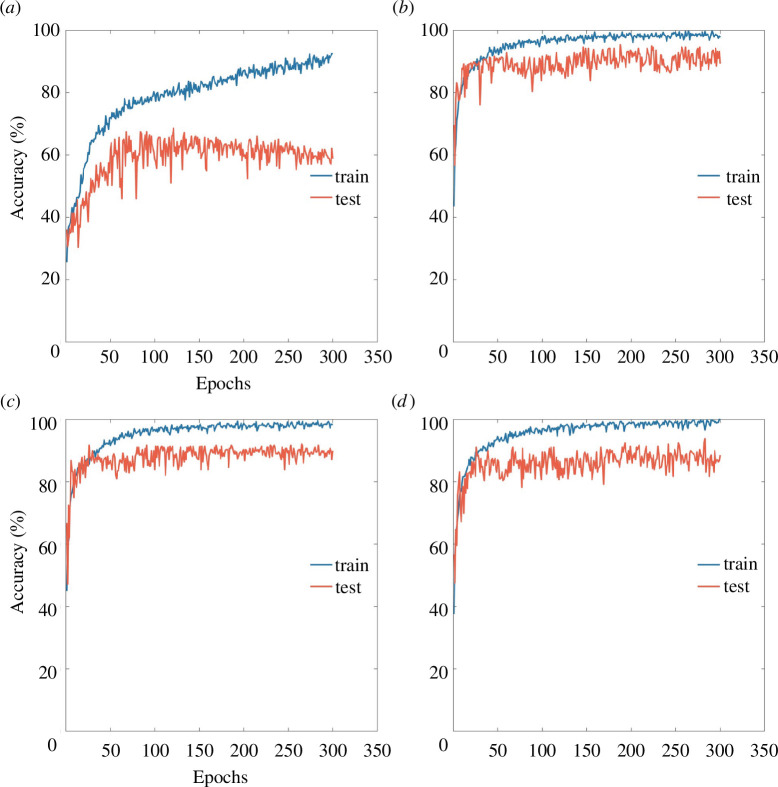
The training process of the last fold in the stratified fivefold cross-validation of the 1D-CNN-12 model. Based on different data inputs: (*a*) the original spectra of black area, (*b*) the original spectra of spot area, (*c*) the original spectra of spot area with SG smoothing and (*d*) the key spectra of spot area with SG smoothing and BOI selection.

Four datasets were used in the ablation experiment, including the original spectra of black area as shown in figure 4*c*, the original spectra of spot area, the original spectra of spot area with SG smoothing, and the key spectra of spot area with SG smoothing and BOI selection. We used spectra from four datasets as inputs to train the networks, with the same one-dimensional CNN structure. The training process and inference results of four networks to test sets are compared in figure 9 and table 2, to illustrate the actual effect of proposed method.

**Table 2.** The training results of four models on the test set.

method	accuracy	index	Doson	Meyeri	Chironides	Milon	Eurypylus	Sarpedon
original spectra of black area	76.00 ± 5.47%	Pr	0.64	0.78	0.88	0.72	0.77	0.77
		Re	0.62	0.64	0.9	0.74	0.8	0.99
		F1	0.63	0.71	0.89	0.73	0.78	0.86
original spectra of spot area	91.64 ± 3.83%	Pr	0.87	0.86	1	0.99	0.84	0.99
		Re	0.79	0.86	1	1	0.98	0.79
		F1	0.83	0.86	1	0.99	0.91	0.88
spot area with SG smoothing	92.36 ± 3.04%	Pr	0.86	0.88	1	0.99	0.86	0.98
		Re	0.82	0.85	1	1	0.96	0.89
		F1	0.84	0.86	1	0.99	0.91	0.93
spot area with BOI selection	89.64 ± 5.09%	Pr	0.82	0.8	1	0.98	0.88	0.87
		Re	0.82	0.76	0.99	1	0.94	0.82
		F1	0.82	0.78	1	0.99	0.91	0.85

### 4.5. Feature regions selection

We can easily find the significant deficiency of non-feature regions for species identification by comparing figure 9*a,b*. The classification accuracy of black area spectra is only 76.00 ± 5.47%, and for some species, it is completely confused, as shown in table 2. Meanwhile, the accuracy of spot area is up to 91.64 ± 3.83%, demonstrating that the ROIs we selected can help improve discriminating ability.

### 4.6. Savitzky–Golay smoothing

With SG smoothing before inputting into the network, the accuracy of ROI spectra is improved to 92.36 ± 3.04%, with smaller variability as shown in figure 9*c*, demonstrating the positive effect of noise suppression.

### 4.7. Feature spectra selection

As shown in table 2, we find that the accuracy of the model based on 47 key spectra is 2.72% lower than that based on full spectra, indicating that the operation of BOI selection has removed some important spectral information. In addition, the amplitude of figure 9*c* is smaller than that of figure 9*d*, and the standard deviation of the key-spectral model is larger. This is mainly owing to the complexity of the spectral features of the samples, which leads to the model being unable to learn effective information when the number of features decreases, resulting in a decrease in classification accuracy and poor model stability [38]. Additionally, we also compared the on-chip inference results of full spectra and feature spectra, and the inference time was improved from 0.6 to 0.35 ms.

The above results confirm our expectations: compared with image-based models, spectral-based models exhibit significant advantages, with SG smoothing operations achieving the highest accuracy of 92.36% and the lowest standard deviation of 3.04%. Although the accuracy of the 1D-CNN-12 model has decreased after using feature wavelengths selection, its performance is still on par with the best image classification results, and its standard deviation performance is superior. When we use spectral information for classification, not only can we improve accuracy, but we can also significantly reduce the number of model parameters and complexity. This means lower computational costs and faster processing speed, which will have a wider range of application scenarios.

## 5. Conclusion

In conclusion, we propose a fast species discrimination method of insects outdoors using HSI and lightweight artificial intelligence algorithm. Feature regions selection, feature spectra selection and model quantification are used for the optimization of discrimination algorithm on chip. As experimental verification of field applications, six butterfly species in the same genus of *Graphium* are selected for rapid discrimination based on the lightweight CNN, which is deployed on a NPU chip. The experimental results show that the spectral classification model corrects the errors of the image classification model in the classification of similar species in Eurypylus and Meyeri. However, on two similar species, Doson and Chironides, the performance of the model did not achieve good results. In terms of overall classification results, the lightweight network extracted from hyperspectral features shows significant advantages, achieving accuracy comparable to machine vision shape recognition in just 0.35 ms of inference time. When we use full spectrum information, we achieve a highly stable and accurate model of 92.36 ± 3.04%. Although the inference time has slightly increased to 0.6 ms, it is still efficient and feasible in practical application scenarios. To further optimize the classification performance of similar species, we plan to combine spatial and spectral information from hyperspectral images in future research, in order to construct more accurate classification models. Our study provides a rapid species discrimination method for similar insects for field use and paves the way for the following research and applications of multi-spectral intelligent sensors based on on-chip spectroscopic microstructure integration.

## Data Availability

Data and relevant description for this work are stored in Dryad repository [39].

## References

[1] Badirli S , Picard CJ , Mohler G , Richert F , Akata Z , Dundar M . 2023 Classifying the unknown: insect identification with deep hierarchical Bayesian learning. Methods Ecol. Evol. **14** , 1515–1530. (10.1111/2041-210X.14104)

[2] Carvajal JA , Romero DG , Sappa AD . Fine-tuning based deep convolutional networks for lepidopterous genus recognition. In 2016 Proc. of the Iberoamerican Congress on Pattern Recognition (CIARP), pp. 467–475. Berlin, Germany: Springer. (10.1007/978-3-319-52277-7_57)

[3] Yasmin R , Das A , Rozario LJ , Islam MdE . 2023 Butterfly detection and classification techniques: a review. Intell. Syst. Appl. **18** , 200214. (10.1016/j.iswa.2023.200214)

[4] Kang SH , Song SH , Lee SH . 2012 Identification of butterfly species with a single neural network system. J. Asia Pac. Entomol. **15** , 431–435. (10.1016/j.aspen.2012.03.006)

[5] Wilson JJ , Karen-Chia HM , Sing KW , Sofian-Azirun M . 2014 Towards resolving the identities of the Graphium butterflies (Lepidoptera: Papilionidae) of Peninsular Malaysia. J. Asia Pac. Entomol. **17** , 333–338. (10.1016/j.aspen.2014.02.007)

[6] Kang SH , Cho JH , Lee SH . 2014 Identification of butterfly based on their shapes when viewed from different angles using an artificial neural network. J. Asia Pac. Entomol. **17** , 143–149. (10.1016/j.aspen.2013.12.004)

[7] Kaya Y , Kayci L . 2014 Application of artificial neural network for automatic detection of butterfly species using color and texture features. Vis. Comput. **30** , 71–79. (10.1007/s00371-013-0782-8)

[8] LeCun Y , Bengio Y , Hinton G . 2015 Deep learning. Nat. New Biol. **521** , 436–444. (10.1038/nature14539) 26017442

[9] Hatcher WG , Yu W . 2018 A survey of deep learning: platforms, applications and emerging research trends. IEEE Access **6** , 24 411–24 432. (10.1109/ACCESS.2018.2830661)

[10] Theivaprakasham H . 2021 Identification of Indian butterflies using deep convolutional neural network. J. Asia Pac. Entomol. **24** , 329–340. (10.1016/j.aspen.2020.11.015)

[11] Nie L , Wang K , Fan X , Gao Y . Fine-grained butterfly recognition with deep residual networks: a new baseline and benchmark. In 2017 Int. Conf. on Digital Image Computing, Sydney, Australia, pp. 1–7. (10.1109/DICTA.2017.8227435)

[12] Kang SH , Jeon W , Lee SH . 2012 Butterfly species identification by branch length similarity entropy. J. Asia Pac. Entomol. **15** , 437–441. (10.1016/j.aspen.2012.05.005)

[13] Khan MJ , Khan HS , Yousaf A , Khurshid K , Abbas A . 2018 Modern trends in hyperspectral image analysis: a review. IEEE Access **6** , 14 118–14 129. (10.1109/ACCESS.2018.2812999)

[14] Goetz AFH , Vane G , Solomon JE , Rock BN . 1985 Imaging spectrometry for earth remote sensing. Science **228** , 1147–1153. (10.1126/science.228.4704.1147) 17735325

[15] Ortega S , Fabelo H , Camacho R , de la Luz Plaza M , Callicó GM , Sarmiento R . 2018 Detecting brain tumor in pathological slides using hyperspectral imaging. Biomed. Opt. Express. **9** , 818–831. (10.1364/BOE.9.000818) 29552415 PMC5854081

[16] Huang H , Qureshi JU , Liu S , Sun Z , Zhang C , Wang H . 2021 Hyperspectral imaging as a potential online detection method of microplastics. Bull. Environ. Contam. Toxicol. **107** , 754–763. (10.1007/s00128-020-02902-0) 32556690

[17] Yu S , Jia S , Xu C . 2017 Convolutional neural networks for hyperspectral image classification. Neurocomputing **219** , 88–98. (10.1016/j.neucom.2016.09.010)

[18] Medina JM , Nascimento SMC , Vukusic P . 2011 Hyperspectral optical imaging of two different species of Lepidoptera. Nanoscale Res. Lett. **6** , 369. (10.1186/1556-276X-6-369) 21711872 PMC3211459

[19] Brydegaard M , Jansson S , Schulz M , Runemark A . 2018 Can the narrow red bands of dragonflies be used to perceive wing interference patterns. Ecol. Evol. **8** , 5369–5384. (10.1002/ece3.4054) 29938059 PMC6010746

[20] Li M , Runemark A , Guilcher N , Hernandez J , Rota J , Brydegaard M . 2023 Feasibility of insect identification based on spectral fringes produced by clear wings. IEEE J. Sel. Top. Quantum Electron. **29** , 1–8. (10.1109/JSTQE.2022.3218218)

[21] Takahashi KH . 2023 Quantitative analysis of wing interference patterns in Drosophila spp. using hyperspectral images. Physiol. Entomol. **48** , 83–89. (10.1111/phen.12405)

[22] Chan WP *et al* . 2022 A high-throughput multispectral imaging system for museum specimens. Commun. Biol. **5** , 1318. (10.1038/s42003-022-04282-z) 36456867 PMC9715708

[23] Sun J , Lu X , Mao H , Wu X , Gao H . 2017 Quantitative determination of rice moisture based on hyperspectral imaging technology and BCC-LS-SVR algorithm. J. Food Process Eng. **40** , e12446. (10.1111/jfpe.12446)

[24] Jin X , Memon H , Tian W , Yin Q , Zhan X , Zhu C . 2017 Spectral characterization and discrimination of synthetic fibers with near-infrared hyperspectral imaging system. Appl. Opt. **56** , 3570–3576. (10.1364/AO.56.003570) 28430236

[25] Islam MN , Nielsen G , Stærke S , Kjær A , Jørgensen B , Edelenbos M . 2018 Novel non-destructive quality assessment techniques of onion bulbs: a comparative study. J. Food Sci. Technol. **55** , 3314–3324. (10.1007/s13197-018-3268-x) 30065443 PMC6045999

[26] Li H , Liang Y , Xu Q , Cao D . 2009 Key wavelengths screening using competitive adaptive reweighted sampling method for multivariate calibration. Anal. Chim. Acta **648** , 77–84. (10.1016/j.aca.2009.06.046) 19616692

[27] Tang N *et al* . 2020 Identification of lycium barbarum varieties based on hyperspectral imaging technique and competitive adaptive reweighted sampling-whale optimization algorithm-support vector machine. J. Food Process Eng. **44** , e13603. (10.1111/jfpe.13603)

[28] Li F , Xiong Y . 2018 Automatic identification of butterfly species based on HoMSC and GLCMoIB. Vis. Comput. **34** , 1525–1533. (10.1007/s00371-017-1426-1)

[29] Schmidt O , Schmidt S , Häuser CL , Hausmann A , Van Vu L . 2019 Using malaise traps for collecting Lepidoptera (Insecta), with notes on the preparation of macrolepidoptera from ethanol. Biodivers. Data J. **7** , e32192. (10.3897/BDJ.7.e32192) PMC6426827 30918447

[30] Freitas AVL *et al* . 2021 Sampling methods for butterflies (lepidoptera). In Measuring arthropod biodiversity: a handbook of sampling methods (eds JC Santos , GW Fernandes ), pp. 101–123. Cham, Switzerland: Springer International Publishing.

[31] Chen H , Qi Z , Shi Z . 2022 Remote sensing image change detection with transformers. IEEE Trans. Geosci. Remote Sens. **60** , 1–14. (10.1109/TGRS.2021.3095166)

[32] Lee E , Lee CY . NeuralScale: efficient scaling of neurons for resource-constrained deep neural networks. In 2020 IEEE/CVF Conf. on Computer Vision and Pattern Recognition (CVPR), Seattle, WA, pp. 1475–1484. (10.1109/CVPR42600.2020.00155)

[33] Koirala A , Walsh KB , Wang Z , McCarthy C . 2019 Deep learning – method overview and review of use for fruit detection and yield estimation. Comput. Electron. Agric. **162** , 219–234. (10.1016/j.compag.2019.04.017)

[34] Gao J , Nuyttens D , Lootens P , He Y , Pieters JG . 2018 Recognising weeds in a maize crop using a random forest machine-learning algorithm and near-infrared snapshot mosaic hyperspectral imagery. Biosyst. Eng. **170** , 39–50. (10.1016/j.biosystemseng.2018.03.006)

[35] Poudel S , Kim YJ , Vo DM , Lee SW . 2020 Colorectal disease classification using efficiently scaled dilation in convolutional neural network. IEEE Access **8** , 99 227–99 238. (10.1109/ACCESS.2020.2996770)

[36] Travers S , Bertelsen MG , Petersen KK , Kucheryavskiy SV . 2014 Predicting pear (CV. Clara Frijs) dry matter and soluble solids content with near infrared spectroscopy. LWT. Food Sci. Technol. **59** , 1107–1113. (10.1016/j.lwt.2014.04.048) 23935002

[37] Wang X , Dong S , Huang S , Wu Q , Fang J , Wang Z , Cheng X . 2023 Near-infrared InGaAs intelligent spectral sensor by 3D heterogeneous hybrid integration. Adv. Photon. Res. **4** , 2300043. (10.1002/adpr.202300043)

[38] Wang Y , Song S . 2023 Variety identification of sweet maize seeds based on hyperspectral imaging combined with deep learning. Infrared Phys. Technol. **130** , 104611. (10.1016/j.infrared.2023.104611)

[39] Wang X . 2024 Data from: Rapid species discrimination of similar insects using hyperspectral imaging and lightweight edge artificial intelligence. Dryad Digital Repository. (10.5061/dryad.cfxpnvxdc) PMC11288683 39086830

